# Distribution and volume of mitochondria in alveolar epithelial type 1 cells in infant and adult human lungs

**DOI:** 10.1007/s00418-024-02332-7

**Published:** 2024-11-18

**Authors:** Arne K. Schierz, Giacomo Rößler, Jan Philipp Schneider, Stefan A. Tschanz, Christopher Werlein, Danny D. Jonigk, Julia Schipke, Christian Mühlfeld

**Affiliations:** 1 Hannover Medical School, Institute of Functional and Applied Anatomy, Carl-Neuberg-Str. 1, 30625 Hannover, Germany; 2 Biomedical Research in Endstage and Obstructive Lung Disease Hannover (BREATH), Member of the German Center for Lung Research (DZL), Hannover, Germany; 3 University of Bern, Institute of Anatomy, Baltzerstrasse 2, 3008 Bern, Switzerland; 4 Hannover Medical School, Institute of Pathology, Carl-Neuberg-Str. 1, 30625 Hannover, Germany; 5 Institute of Pathology, University Clinics of RWTH University, Aachen, Germany

**Keywords:** Alveolar epithelium, Mitochondria, Alveolar epithelial type 1 cells, Development, Stereology

## Abstract

Alveolar epithelial type I (AE1) cells with their wide spatial expansion form approximately 95% of the outer surface area of the air-blood barrier inside the lung. Serial block-face scanning electron microscopy (SBF-SEM) investigations led to the hypothesis that AE1 cell mitochondria are preferentially distributed as aggregates in those parts of AE1 cells that are located above connective tissue pillars between capillaries, thus not increasing the thickness of the diffusion distance for oxygen and carbon dioxide. Furthermore, it was hypothesised that postnatal development requires adapting the amount and distribution of mitochondria in AE1 cells. Human lung samples from three infant (26 and 30 days, 6 months) and three adult (20, 39 and 40 years) samples were investigated by light microscopy, transmission electron microscopy and stereology. The volume fraction of mitochondria was similar in infant and adult lungs with a mean value of 6.3%. The ratio between mitochondrial profiles on top of capillaries or above connective tissue pillars was approximately 3:1 in infants and adults. However, regarding the volume of both cytoplasmic compartments, infants showed a higher number of mitochondrial profiles on top of capillaries while adults showed a higher number above connective tissue pillars. Samples of three additional adult lungs were analysed by confocal laser scanning microscopy. Again, mitochondria were not preferentially found as aggregates above connective tissue pillars. In conclusion, AE1 cell mitochondria were not preferentially found as aggregates, showed the same volume density in infants and adults but differed in distribution between the age groups.

## Introduction

The alveolar epithelium of the mammalian lung consists of alveolar epithelial type 1 (AE1) and type II (AE2) cells. While the surfactant-producing AE2 cells have a more or less cuboidal shape that reside in niches of alveoli (Schneider et al. 2020), AE1 cells possess enormous cytoplasmic extensions protruding from the nucleus-containing cell body (Weibel 2015). These extensions form the major part of the outer cellular layer of the air-blood barrier, which has a 2.2 µm thickness on average and only 0.2 µm in its thinnest parts in healthy adult human lungs (Gehr et al. 1978). The thin barrier separating air and blood permits efficient diffusion of oxygen and carbon dioxide. AE1 cells form a relatively tight epithelium and possess various transport proteins for ion and water, which contribute to the homeostasis of the thin layer of fluid on top of the alveolar epithelium, the so-called hypophase (Weibel and Gil 1968; Hollenhorst et al. 2011). To keep the air-blood barrier thin, the organelles of AE1 cells like the nucleus and mitochondria were described to be located mostly in parts of the cell that are not underpinned by capillaries and therefore do not contribute to the thickness of the air-blood barrier in its narrower sense (Weibel 1973). On the other hand, this has the disadvantage that energy-rich substrates like ATP have a prolonged diffusion distance to peripheral parts of the cells to fulfill the needs of water and ion transport.

Infant human lungs exhibit morphological differences compared to adult lungs, e.g. the arithmetic mean thickness of the postnatal (approximately 1 month) infant air-blood barrier is 5 µm and then decreases to half of this value during the first 5 years of life (Zeltner et al. 1987). Alveolarization and microvascular maturation are not completed until birth but continue until young adulthood (Schmid et al. 2023). The total number of alveoli, which usually is < 50 million at birth, increases to > 400 million in adulthood (Ochs et al. 2004). For the formation of alveoli, secondary septa grow out from primary septa, which initially show a double-layered capillary network. A fusion of the two layers of capillaries and a flattening of AE1 cells take place by reciprocal crosstalk between airway epithelium and vascular endothelium to form the mature interalveolar septa and a thinner air-blood barrier (Schittny 2017).

**Fig. 1:**
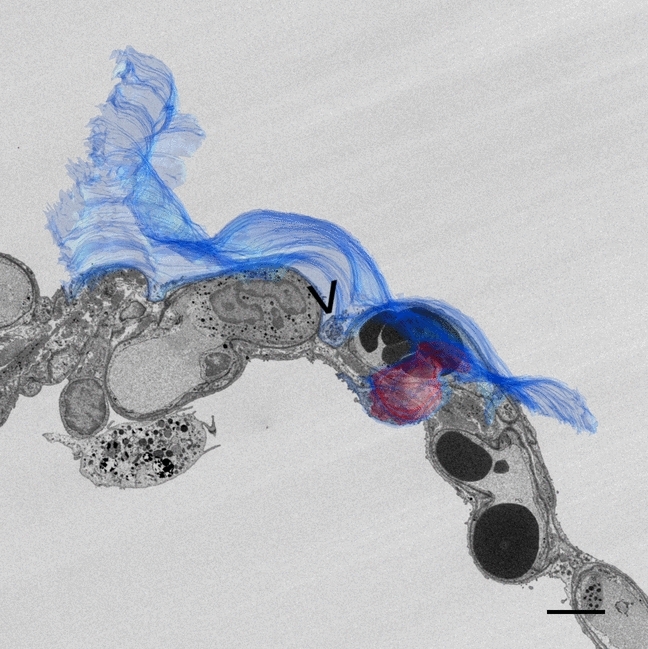
Serial block-face scanning electron micrograph of an interalveolar septum of an adult human lung. The figure shows a merge of an electron micrograph taken from a data set generated by SBF-SEM and a transparent model of an alveolar epithelial type I cell segmented from this data set (blue: cell body, red: nucleus). The arrowhead marks a cytoplasmic bulge of the AE1 cell with a cluster of mitochondria. This cluster is located in a niche of the alveolar capillary network between two capillary segments. The cytoplasm on top of the surrounding capillaries remains thin, keeping the air-blood barrier thin. The underlying data set, a similar electron micrograph and the reconstruction of these alveolar epithelial type I cells were part of another study and have been published elsewhere (Schneider et al. 2019). Adapted with permission from the American Thoracic Society. Copyright © 2023 American Thoracic Society. All rights reserved. Cite: Schneider JP, Wrede C, Hegermann J, Weibel ER, Mühlfeld C, Ochs M. 2019. On the Topological Complexity of Human Alveolar Epithelial Type 1 Cells. Am J Respir Crit Care Med. 199:1153–1156. The American Journal of Respiratory and Critical Care Medicine is an official journal of the American Thoracic Society. Readers are encouraged to read the entire article for the correct context at [https://www.atsjournals.org/doi/full/10.1164/rccm.201810-1866LE]. The authors, editors and The American Thoracic Society are not responsible for errors or omissions in adaptations. The mitochondrial finding has been presented at the 7th annual meeting of the German Center for Lung Research (Schneider et al. 2018a) and the 113th annual meeting of the Anatomische Gesellschaft (Schneider et al. 2018b)

Recently, serial block-face scanning electron microscopy (SBF-SEM) was used to perform 3D reconstructions of entire AE1 cells by segmentation of consecutive block-face images and subsequent modelling (Schneider et al. 2019). In the process of that study, some mitochondria were found to form small clusters in the cell periphery, for example between capillary segments (Fig. 1), where the interference with gas exchange should be low, suggesting an optimized structural design enabling efficient energy supply combined with a thin air-blood barrier (Schneider et al. 2018a).

Therefore, this study investigated the distribution and localization of mitochondria in AE1 cells in relation to AE1 cell nuclei and the underlying capillaries in adult and infant human lungs. Specifically, the following hypotheses were tested: (1) AE1 cell mitochondria are preferentially located as aggregates in regions playing a minor role in gas exchange (above connective tissue pillars). (2) During alveolarization and the associated remodelling of AE1 cells, the amount and distribution of AE1 cell mitochondria change and therefore differ between childhood and adulthood. These hypotheses were tested using a combination of transmission electron microscopy (TEM), confocal laser scanning microscopy (CLSM) and stereology.

## Materials and methods

### Human lung tissue

The electron microscopic and stereological analyses were performed on samples of archived lung tissue originally prepared for the studies by Zeltner et al. (1987) (infants) and Gehr et al. (1978) (adults). Fixation and use of the lungs were done according to the bioethical regulations of the University of Bern at the respective time. The use of the archived material was approved by the ethics committee of Hannover Medical School (Permission No. 2263–2014). The subjects from which the samples were taken had died from non-pulmonary causes. Three infant (C1, 26 days; C2, 30 days; C3, 6 months) and three adult (A2, 40 years; A4, 20 years; A8, 39 years) lungs of the originally reported lungs were picked for this study.

After death, lungs were fixed by instillation via the airways using a fixative containing 2.5% glutaraldehyde in phosphate buffer. The samples used in this study were subsequently incubated with osmium tetroxide and uranyl acetate before dehydration and embedding in epoxy resin. For further details, see Zeltner and Burri (1987), Zeltner et al. (1987) and Gehr et al. (1978). Semi- and ultrathin sections (60–80 nm) were obtained from 6–7 epoxy resin-embedded samples per individual. Semithin sections for conventional light microscopy were stained with toluidine blue and ultrathin sections were stained with lead citrate/uranyl acetate.

For CLSM, lung samples either from tumour far tissue of tumour resection specimens or downsizing material during lung transplantation of three patients were obtained from the Institute of Pathology at Hannover Medical School (ethics vote number: 1741-2013 and 3381-2016). Tissue was fixed in 4% buffered formaldehyde directly after surgery and afterwards embedded in paraffin.

### Design-based stereology

For estimation of stereological parameters well-established guidelines and recommendations were used (Hsia et al. 2010; Ochs and Mühlfeld 2013). The volume fraction of interalveolar septa was determined using light microscopic images and a point grid consisting of a coarse and a fine grid. Images were obtained using a Zeiss AxioScan Z.1 slide scanner (Zeiss, Göttingen, Germany) and analysed using the Visiopharm stereology software (Visiopharm, Hørsholm, Denmark). The coarse grid, consisting of four points, was used to count points hitting air in gas exchanging parts of the lung. The fine grid, consisting of 24 points, was used to count points hitting interalveolar septa. All fields of view were obtained by systematic uniform random sampling (SURS) at an objective lens magnification of 20 × (Gundersen and Jensen 1987). The volume fraction of interalveolar septa regarding the parenchyma [V_V_ (sept/par)] was calculated by dividing the number of points hitting interalveolar septa by the number of points hitting interalveolar septa and air multiplied by six. The volume fractions of AE1 cells and their mitochondria were determined by TEM (Morgagni, FEI, Eindhoven, The Netherlands). A point grid consisting of a coarse and fine grid was projected on fields of view obtained by SURS at a primary magnification of 8900 × using the STEPanizer (Tschanz et al. 2011). The coarse grid consisted of nine points and was used to count points hitting AE1 cells and the interalveolar septum. The fine grid (144 points) was used to count points hitting AE1 cell mitochondria. The volume fraction of AE1 cells per unit volume of interalveolar septum [V_V_ (AE1/sept)] was determined by dividing the number of points hitting AE1 cells by the number of points hitting the interalveolar septum. The volume fraction of mitochondria per unit volume of AE1 cells was determined by dividing the number of points hitting mitochondria by the number of points hitting AE1 cells multiplied by 16. To calculate the total volumes of interalveolar septa, AE1 cells and AE1 cell mitochondria, the fractions were multiplied by the respective reference volume. Therefore, the total volume of parenchyma was needed, which was taken from the data published by Zeltner et al. (1987). Also, the fractions of cross-sectional mitochondrial profiles Q (ger.: “Querschnitt”) in the compartments defined below were determined. For determination of Q no test system was used, but every profile was counted (Sterio 1984).

For subsequent distribution analysis a modification of a method published by Mühlfeld et al. (2007) was used. Two compartments of AE1 cells were defined: (1) AE1 cell portions on top of capillaries, i.e. in the main parts of the gas-exchange interface and (2) AE1 cell portions above connective tissue pillars, i.e. playing a minor role in gas exchange. In short, an estimator for a compartment size was compared to the number of mitochondrial profiles in this compartment. Therefore, a point grid with 25 points was used to determine the number of points P hitting either the first or the second compartment defined above. Also, the number of mitochondrial profiles (N_0_) in each of the two compartments was counted. With these data, the number of expected mitochondrial profiles (N_E_) in each compartment was calculated based on the assumption of random distribution. Subsequently, an index of relative localization (IRL) was determined by dividing N_0_ by N_E_ as an expression for random (IRL≈1), preferential (IRL > 1) or non-preferential (IRL < 1) localization. Chi-squared analysis was used to test the null hypothesis that “the observed and expected distributions of mitochondrial profiles are equal”.

### Immunohistochemistry

For qualitative evaluation and quantitative colocalization studies of mitochondria in 10-µm-thick paraffin sections of AE1 cells of three adult human lungs were immunolabelled with (1) advanced glycosylation end product-specific receptor (AGER) antibody, (2) translocase of outer mitochondrial membrane-20 (TOMM-20) antibody and (3) DAPI to visualize (a) AE1 cell plasma membrane, (b) mitochondria and (c) nuclei, respectively. Fehrenbach et al. showed that AGER antibodies are specific for the basolateral membrane of AE1 plasma membranes in rat and human lung tissue by immunohistochemistry, double immunofluorescence and immunoelectron microscopy (Fehrenbach et al. 1998). For recombinant monoclonal TOMM-20 antibodies we relied on the manufacturer's (Abcam, Cambridge, UK) statement concerning the specificity of recombinant antibodies. In short, tissue was deparaffinized in xylene with decreasing isopropyl alcohol proportions. Sections were washed with distilled water, incubated twice in Dako retrieval buffer pH 6.0 (Dako, Glostrup, Denmark) at 700 W seven mins, cooled on ice for 30 min, washed with distilled water and incubated again with 5% donkey serum (Dianova, Hamburg, Germany), 1% bovine serum albumin (BSA; Serva, Heidelberg, Germany) and 0.3% Triton X-100 (TX-100; Sigma-Aldrich, Steinheim, Germany) in PBS for 60 min at room temperature. After washing, sections were incubated with polyclonal goat anti-AGER antibody (Bio-Techne, Wiesbaden, Germany; order number: AF1145) diluted 1:100 in PBS containing 1% BSA and 0.3% TX-100 over night. Then, sections were incubated with Alexa647-linked donkey-anti-goat antibody diluted 1:1000 in PBS (Thermo Fisher Scientific, Waltham, MA, USA), 1% BSA and 0.3% TX-100 for 60 min. Afterwards, the sections were incubated with monoclonal rabbit anti-TOMM-20 antibody (Abcam, Cambridge, UK; order number: ab186735) diluted 1:250 in PBS containing 1% BSA, 0.3% TX-100 and 5% goat serum (Biozol, Eching, Germany). Subsequently, the sections were transferred into PBS containing 1:1000 Alexa488-linked goat-anti-rabbit antibody (Thermo Fisher Scientific, Waltham, MA, USA), 1% BSA and 0.3% TX-100. Before and after each individual antibody containing step, the sections were washed with PBS-Tween first for 5 and then for 10 min. Finally, the sections were transferred into PBS containing 1:1000 DAPI (Thermo Fisher Scientific, Waltham, MA, USA) for 15 min and washed with PBS first for 5 and then 10 min. Sections were embedded in Mowiol 4–88 (Carl Roth, Karlsruhe, Germany) and sealed with a cover slip.

#### CLSM

Immunohistochemically stained specimens of human lungs were investigated using a Zeiss LSM 980 microscope (Zeiss, Jena, Germany). Three sites in each specimen were investigated by taking an overview image and consecutive imaging along the z-axis (z-stacks). Z-stacks were generated on parts of the section that contained interalveolar septa and visible mitochondria. Z-stacks were recorded at 63 × objective lens magnification with a crop factor of 8 or 16 (final size: 26.52 µm × 26.52 µm × 10 µm or 13.26 µm × 13.26 µm × 10 µm) to avoid areas without interalveolar septa. Parameters like pin hole and z-distance were optimized to obtain z-stacks of 10 µm thickness and maximal resolution. The z-stacks and the software Imaris (Imaris × 64, version 8.2.1, Bitplane, Belfast, UK) were used to generate 3D reconstructions. The 3D-derived reconstructions were confirmed by a colocalization analysis of AE1 cell mitochondria and AE1 cell membrane based on the signals of Alexa488-linked mitochondria and Alexa647-linked AE1 cell membrane. The colocalization analysis was processed with the JACoP plugin (Bolte and Cordelières 2006) for Fiji software (Schindelin et al. 2012). We assumed that, due to the highest resolution of CLSM of approximately 0.2 µm (Elliott 2020) and the thin cytoplasmic extensions of AE1 cells with a thickness of 0.2–0.1 µm (Weibel 1971; Dobbs et al. 2010), mitochondria inside AE1 cells are colocalized with the basolateral AE1 cell plasma membrane. As reproducible colocalization parameters, Pearson’s coefficient (PC) with Costes’ automatic threshold (Costes et al. 2004) and the local maximum in Van Steensel’s cross-correlation coefficient (CCF) distribution (van Steensel et al. 1996) were used. Three cases of parameters were evident: (1) negative PC and local CCF maximum far from zero, (2) positive PC and local CCF maximum (almost) zero and (3) positive PC and local CCF maximum far from zero, therefore, showing (1) no colocalization, (2) colocalization and (3) partial colocalization. Furthermore, we classified the value of PC with a classification of Evans by (1) no, (2) weak and (3) very weak (Evans 1996).

## Results

### Distribution of mitochondria in AE1 cells

**Fig. 2:**
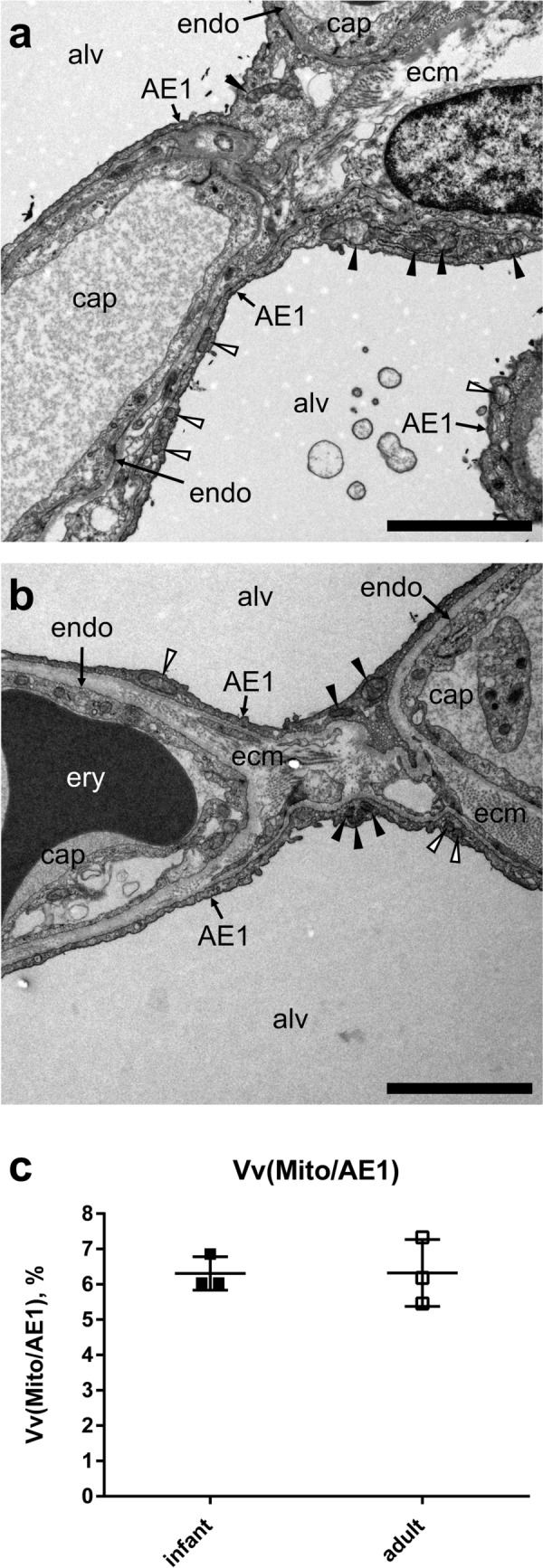
Ultrastructure of AE1 cells and volume density of AE1 cell mitochondria. Transmission electron micrographs of interalveolar septa of infant (**a**) and adult (**b**) lung. Mitochondria located above connective tissue pillar are labelled by black arrowheads, mitochondria located on top of capillaries with white arrowheads. **c** Volume fraction of mitochondria in AE1 cells for infant and adult lungs. endo = capillary endothelium, ecm = extracellular matrix, alv = alveolar lumen, cap = capillary lumen, ery = erythrocyte, scale bar = 3 µm

Mitochondria in infant and adult AE1 cells were often found along the air-blood barrier in the thin AE1 cell extensions, frequently in perinuclear regions and scarcely as clusters in AE1 cell niches between capillary segments. Although the latter were observed (Fig. 2) their occurrence was very rare. The ratio between mitochondrial profiles (counted using TEM) on top of capillaries or above connective tissue pillars was approximately 3:1 in infants and adults (Table 1). However, the contribution of the volume of the two cellular compartments to AE1 cell volume was different in infant and adult lungs with a higher volume fraction of AE1 cell on top of capillaries in adults than in infants. Conversely, the volume fraction of AE1 cell above connective tissue pillars was higher in the infant than adult lungs. Consequently, analysis of IRL with subsequent chi-squared analysis indicates that AE1 cell mitochondria in infants (Table 2) are more frequently located along gas exchange regions than expected from the volume of this compartment. In adults (Table 3), the mitochondria were more frequently located in regions above connective tissue pillars than expected from the volume of this cellular compartment.

**Table 1:** Overview on the stereological data

	Infant			Adult		
Parameter	C1	C2	C3	A2	A4	A8
V(par), cm^3^	115	157	372	3285	3510	5355
V_V_(sept/par)	0.325	0.288	0.143	0.085	0.198	0.114
V(sept, par), cm^3^	37.3	45.3	53.1	279.0	695.0	612.3
V_V_(AE1/sept)	0.154	0.117	0.105	0.083	0.107	0.107
V(AE1, sept), cm^3^	5.7	5.3	5.6	23.0	74.3	65.6
V_V_(Comp1/AE1)	0.613	0.494	0.557	0.691	0.748	0.749
V(Comp1, AE1), cm^3^	3.5	2.6	3.1	15.9	55.6	49.1
V_V_(Comp2/AE1)	0.273	0.391	0.367	0.217	0.174	0.181
V(Comp2, AE1), cm^3^	1.6	2.1	2.1	5.0	12.9	11.9
V_V_(Nuc/AE1)	0.114	0.115	0.076	0.092	0.078	0.070
V(Nuc, AE1), cm^3^	0.6	0.6	0.4	2.1	5.8	4.6
V_V_(Mito/AE1)	0.060	0.069	0.060	0.073	0.055	0.062
V(Mito, AE1), cm^3^	0.347	0.364	0.337	1.690	4.055	4.056
Q(Mito, Comp1), %	76.7	74.9	75.6	71.7	76.3	71.5
Q(Mito, Comp2), %	23.3	25.1	24.4	28.3	23.7	28.5

V(par) was taken from Zeltner et al. 1987 (infants) and Gehr et al. 1978 (adults). V(par): parenchymal volume, V_V_(sept/par): volume fraction of interalveolar septa in parenchyma, V(sept, par): total volume of interalveolar septa, V_V_(AE1/sept): volume fraction of AE1 cells in interalveolar septa, V(AE1, sept): total volume of AE1 cells, V_V_(Comp1/AE1): volume fraction of the compartment on top of capillaries in AE1 cells, V(Comp1, AE1): total volume of the compartment on top of capillaries in AE1 cells, V_V_(Comp2/AE1): volume fraction of the compartment above connective tissue pillars excluding the nucleus in AE1 cells, V(Comp2, AE1): total volume of the compartment above connective tissue pillars excluding the nucleus in AE1 cells, V_V_(nuc/AE1): volume fraction of nuclei in AE1 cells, V(nuc, AE1): total volume of nuclei in AE1 cells, V_V_(Mito/AE1): volume fraction of mitochondria in AE1 cells, V(Mito, AE1): total volume of mitochondria inside AE1 cells, Q(Mito, Comp1): fraction of mitochondrial profiles in the compartment on top of capillaries, Q(Mito, Comp2): fraction of mitochondrial profiles in the compartment above connective tissue pillars

**Table 2:** Chi-squared analysis of mitochondria distribution in infant specimens

AE1 compartment	Number of observed mitochondrial profiles, N_0_	Number of observed points, P	Number of expected mitochondrial profiles, N_E_	Index of relative localization, IRL	Chi-squared values
On top of capillaries	1374	1841	1084.77	1.27	77.12
Above connective tissue pillars	442	1241	731.23	0.60	114.40
Total	1816	3082	1816	1.87	191.52

With 1 degree of freedom, the total chi-squared value of 191.52 indicates that the null hypothesis must be rejected (*p* < 0.001). Therefore, mitochondria inside infant AE1 cells are rather located in the region of gas exchange

**Table 3:** Chi-squared analysis of mitchondria distribution in adult specimens

AE1 compartment	Number of observed mitochondrial profiles, N_0_	Number of observed points, P	Number of expected mitochondrial profiles, N_E_	Index of relative localization, IRL	Chi-squared values
On top of capillaries	859	1306	924.39	0.93	4.63
Above connective tissue pillars	311	347	245.61	1.27	17.41
Total	1170	1653	1170	2.20	22.04

With 1 degree of freedom, the total chi-squared value of 22.04 indicates that the null hypothesis must be rejected (*p* < 0.001). Therefore, mitochondria inside adult AE1 cells are rather located in the region between capillaries/perinuclear

### Aggregates of mitochondria in AE1 cells

A total of 1779 TEM images from infant lungs and 1054 TEM images from adult lungs were obtained by SURS. Only 14 images in the infant group and 15 images in the adult group showed aggregates of mitochondria without revealing an immediate proximity to the nucleus.

**Fig. 3:**
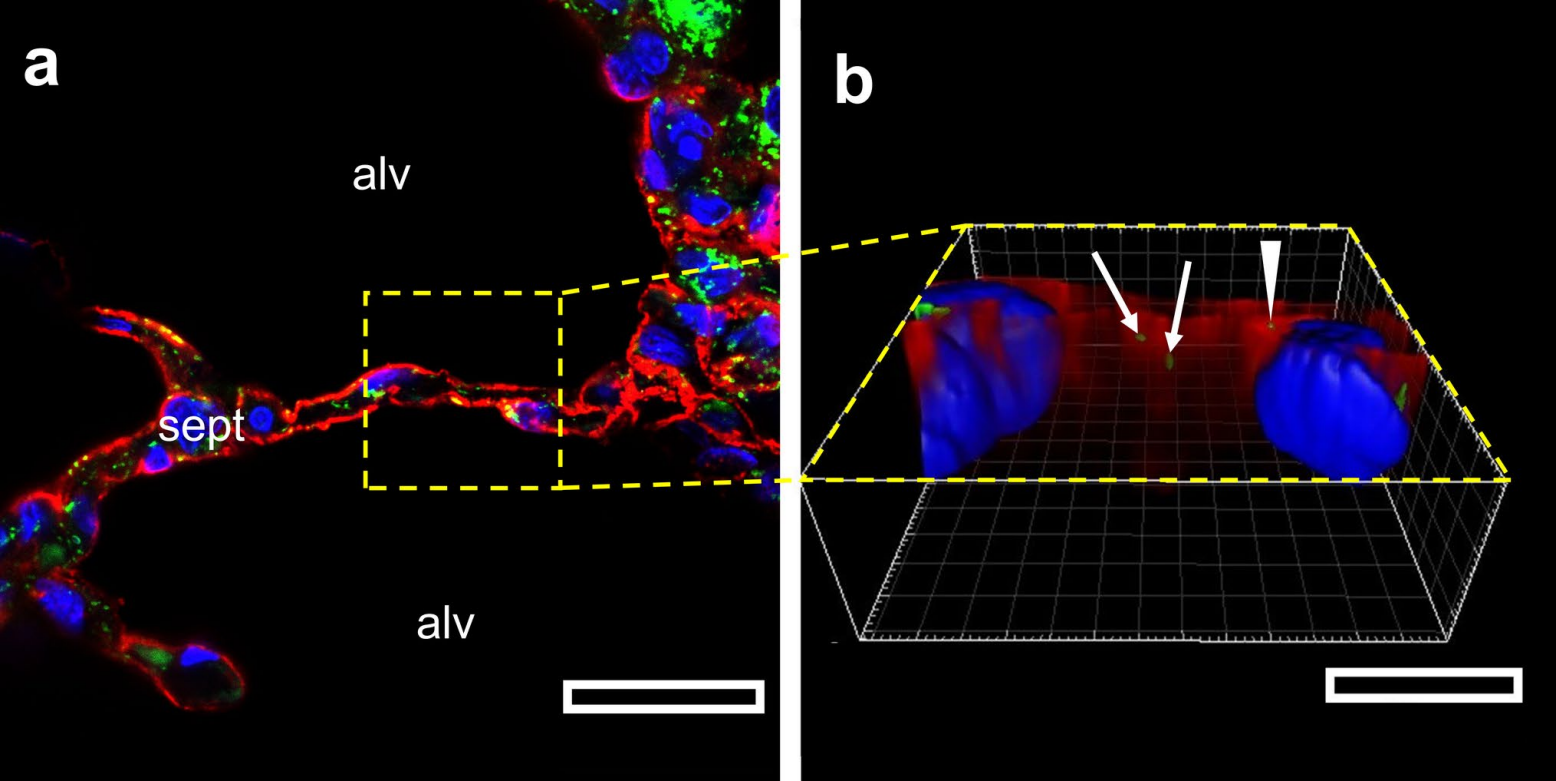
CLSM of mitochondria and AE1 cells in human lung. The signals of AE1 cell basolateral plasma membrane are labelled in red, of mitochondria in green and of nuclei in blue. In (**a**) an uncropped image (lens magnification = 40×, scale bar = 25 µm) of a colocalization between mitochondria and basolateral membrane of AE1 cell is shown. The yellow rectangle depicts the area used to record the corresponding z-stack used for 3D reconstruction (**b**, scale bar = 10 µm). Here, three mitochondria are visible associated with the AE1 cell plasma membrane, two of them in the center of the 3D reconstruction (white arrows) and one next to the nucleus (white arrowhead). Two additional mitochondria are not colocalized with the AE1 cell basolateral plasma membrane and therefore not part of an AE1 cell

In CLSM, the epithelium of interalveolar septa was identified using the fluorescence signal of the basolateral plasma membrane of AE1 cells labelled with AGER antibodies (Shirasawa et al. 2004). Discontinuities in this red signal along the septa can be explained by the presence of unlabelled AE2 cells. Inside the interalveolar septa mitochondria and nuclei were labelled with a TOMM-20 antibody or DAPI, respectively. Mitochondria (green signal) were distributed heterogeneously in the interalveolar septa and frequently near nuclei (blue signal). An exemplary uncropped CLSM image with 3D reconstructions is shown in Fig. 3. In the case of colocalization mitochondria were found inside the labelling of the AE1 plasma membrane, while in case of no colocalization mitochondria were found inside the septum. In all nine investigated sites, we were not able to find any further mitochondrial aggregates inside AE1 cells, thus supporting the TEM result that mitochondrial aggregates are a rather rare event in AE1 cells.

### Quantitative postnatal development of mitochondria in AE1 cells

Table 1 shows a summary of the stereological data. Volume fractions and total volumes of interalveolar septa and AE1 cells were obtained as reference volumes for calculation of total mitochondrial volumes. The volume fraction of mitochondria in AE1 cells showed the same mean value for infant (V_V_ (Mito/AE1) = 6.3%) and adult lungs (V_V_ (Mito/AE1) = 6.3%). Furthermore, the infant lungs showed a similar variation with values of 6.0%, 6.0% and 6.9% in comparison to the adult lungs with 5.5%, 6.2% and 7.3%. Parallel to postnatal lung growth, the total volume of AE1 cell mitochondria increased from approximately 0.35 cm^3^ in the infant lungs to > 4 cm^3^ in two of the adult lungs.

## Discussion

The present study tested the hypotheses that (1) AE1 cell mitochondria are preferentially located as aggregates in regions playing a minor role in gas exchange (above connective tissue pillars) and (2) that the amount and distribution of mitochondria differs between infant and adult human lungs.

In contrast to their progenitors, the AE2 cells, AE1 cells cover the major part of the alveolar region of the mammalian lung (Crapo et al. 1982). The design of this surface area is to provide a huge diffusion area and a minimal diffusion distance between air and blood (Gehr et al. 1981; Weibel 2017). Thus, the protrusions of AE1 cells mainly consist of two sheets of plasma membrane with a small amount of cytoplasm in between. This morphology led to their description of non-nucleated plates before the era of electron microscopy (Weibel 1971; Ochs et al. 2016). Every organelle located in these parts of the cells increases the thickness of the air-blood barrier and decreases the diffusion capacity. However, AE1 cells also have metabolic tasks, such as water and ion transport, that require energy supply along the entire cell (Dobbs et al. 2010). Thus, the mere distribution of mitochondria-generated ATP from the perinuclear region to the most distal parts of the cell might be the best design from the perspective of gas exchange but might be insufficient to meet the metabolic demands of the cell. Both two-dimensional analyses and 3D reconstructions of whole AE1 cells led to the hypothesis that mitochondria form aggregates in cellular niches between capillary segments. Such mitochondrial aggregates could serve as power stations in the more peripheral parts of the cells to decrease the diffusion distance of ATP to the cellular periphery (Schneider et al. 2018a).

The size of the AE1 cell mitochondria profiles is relatively small, approximately in the range of 200–500 nm. TEM is ideally suited to visualize the AE1 cell mitochondria because of its high resolution but fails to provide the necessary 3D information (Schneider et al. 2021). Thus, it is usually impossible to say whether a certain mitochondrial cluster lies in the periphery or the perinuclear region of the AE1 cell. At the electron microscopic level, serial block-face and focused ion beam scanning electron microscopy provides high resolution in combination with 3D information but has other characteristics that limit its use in the current situation (e.g. inverse relationship between resolution and volume, high cost of time and computer capacity) (Schneider et al. 2021). CLSM has the disadvantage that the mitochondrial size as well as the thickness of the AE1 cell processes is at the limit of the microscopic resolution and requires labelling of specific targets without offering an open view (Ochs et al. 2016; Schneider et al. 2021). However, it helps to analyse larger samples in all three dimensions, in a convenient frame of time, and fluorescent labels allow the use of automatic segmentation tools. Due to the various advantages and disadvantages, a combined approach of different techniques was chosen. As design-based stereology rests on a solid mathematical background and provides unbiased quantitative data in 3D (Knudsen et al. 2021), it was used to analyse whether the maturation of AE1 cells during development from childhood to adulthood affects the volume and distribution of AE1 cell mitochondria.

In contrast to the first hypothesis, the present study does not support the concept of a preferential localization of mitochondria as aggregates above connective tissue pillars. The CLSM analysis mostly showed separate mitochondria in the peripheral thin parts of AE1 cells. The relative number of mitochondrial profiles in TEM in the perinuclear region or as aggregates in the periphery accounted for approximately 25% in all investigated lungs, irrespective of the developmental status. Perinuclear mitochondria and mitochondria above connective tissue pillars were defined as a common compartment, because without further 3D information it cannot be unambiguously identified whether a mitochondrion is in close proximity to the nucleus from a single ultrathin section. It was striking that three quarters of the mitochondrial profiles were located in the thin part of the AE1 cells, yet this result does not consider the volume fractions of the two compartments. However, the chi-squared analysis, regarding the different volume fractions of the AE1 cytoplasm, showed that differences between infants and adults were present that may suggest a developmental shift of mitochondria away from the air-blood barrier in adult lungs. One can conclude that the metabolic functions of AE1 cells require short distances for diffusion of ATP to the sites of demand—a mere localization as aggregates in niches of cells does not seem to be favorable. Besides their function as producers of ATP, the signalling functions of peripheral AE1 cell mitochondria during postnatal development might play a role, like generation of reactive oxygen species (ROS) by mitochondria, which is followed by VEGF upregulation and finally lead to angiogenesis (Lee et al. 2020). Mercurio and Rhodin (1984) performed 3D reconstructions of one AE1 cell each in neonatal, 14 days old and 90 days old cats. They described that the number of mitochondria per cell increased successively throughout postnatal development. In addition, they showed that at birth mitochondria were mainly localized at thin cytoplasmic extensions and later, at 14 day, they were also found perinuclearly. This postnatal change in mitochondrial distribution is in good agreement with the changes of IRL from the infant to adult group in this study when considering that the postnatal feline lung development already shows formation of secondary septa during the first 14 days (Al‐Tikriti et al. 1991).

In the second hypothesis it was proposed that the number of AE1 cell mitochondria changes during postnatal human lung development. Given the profound changes taking place during postnatal lung development this hypothesis was reasonable. It is well known that alveolarization is accompanied by microvascular maturation, i.e. the reduction of the double-layered capillary network of the interalveolar septa to a single layer (Schmid et al. 2023; Zeltner and Burri 1987) and that during this phase the thickness of the air-blood barrier decreases (Zeltner et al. 1987; Schmid et al. 2023). It was shown that the reduction of the air-blood barrier thickness is mainly due to a reduction of AE1 cell volume as shown by the decreasing ratio of AE1 and AE2 cell volume (Vidić and Burri 1983). However, the stereological data obtained in this study show that the volume density of AE1 cell mitochondria is very similar in infant and adult human lungs. Interestingly, the mitochondrial volume density reported in this study is close to the one described in the adult mouse and rat lung by Massaro et al. (1975). Thus, it can be concluded that AE1 cells have a relatively constant volume fraction of mitochondria throughout postnatal development and across species. Of course, the total volume of AE1 cell mitochondria rises during development as do the total volume of AE1 cells and lung volume. However, the relationship between AE1 cell volume and mitochondria seems to be well controlled.

In summary, there was no evidence of a preferential localization of AE1 cell mitochondria as aggregates in the niches of AE1 cells between capillaries. The ratio of single mitochondrial profiles on top of capillaries to those clustered and/or above connective tissue pillars was approximately 3:1 in both infant and adult lungs. However, chi-squared analysis regarding the volume of the investigated compartments revealed a slightly but significantly preferred localization of AE1 cell mitochondria on top of capillaries in infants and above connective tissue pillars in adults. Also, the volume fraction of mitochondria remained constant throughout postnatal development indicating that already in the infant lung the metabolic tasks of AE1 cells require a constant supply by mitochondria in all parts of the cell.

## Data Availability

No datasets were generated or analysed during the current study.

## References

[CR1] Al-Tikriti MS, Henry RW, Eiler H et al (1991) Fine structural aspects of postnatal development of feline lung. Anat Histol Embryol 20:311–319. 10.1111/j.1439-0264.1991.tb00306.x 1796783 10.1111/j.1439-0264.1991.tb00306.x

[CR2] Bolte S, Cordelières FP (2006) A guided tour into subcellular colocalization analysis in light microscopy. J Microsc 224:213–232. 10.1111/j.1365-2818.2006.01706.x 17210054 10.1111/j.1365-2818.2006.01706.x

[CR3] Costes SV, Daelemans D, Cho EH et al (2004) Automatic and quantitative measurement of protein-protein colocalization in live cells. Biophys J 86:3993–4003. 10.1529/biophysj.103.038422 15189895 10.1529/biophysj.103.038422 PMC1304300

[CR4] Crapo JD, Barry BE, Gehr P et al (1982) Cell number and cell characteristics of the normal human lung. Am Rev Respir Dis 126:332–337. 10.1164/arrd.1982.126.2.332 7103258 10.1164/arrd.1982.126.2.332

[CR5] Dobbs L, Johnson M, Vanderbilt J et al (2010) The great big alveolar TI cell: evolving concepts and paradigms. Cell Physiol Biochem 25:055–062. 10.1159/000272063 10.1159/000272063 20054144

[CR6] Elliott AD (2020) Confocal microscopy: principles and modern practices. Curr Protoc Cytom. 10.1002/cpcy.68 31876974 10.1002/cpcy.68 PMC6961134

[CR7] Evans JD (1996) Straightforward statistics for the behavioral sciences. Duxburry Press

[CR8] Fehrenbach H, Kasper M, Tschernig T, et al (1998) Receptor for advanced glycation endproducts (RAGE) exhibits highly differential cellular and subcellular localisation in rat and human lung. Cell Mol Biol (Noisy-le-grand) 44:1147–57 9846897

[CR9] Gehr P, Bachofen M, Weibel ER (1978) The normal human lung: ultrastructure and morphometric estimation of diffusion capacity. Respir Physiol 32:121–140. 10.1016/0034-5687(78)90104-4 644146 10.1016/0034-5687(78)90104-4

[CR10] Gehr P, Mwangi DK, Ammann A et al (1981) Design of the mammalian respiratory system. V. Scaling morphometric pulmonary diffusing capacity to body mass: wild and domestic mammals. Respir Physiol 44:61–86. 10.1016/0034-5687(81)90077-3 7232887 10.1016/0034-5687(81)90077-3

[CR11] Gundersen HJG, Jensen EB (1987) The efficiency of systematic sampling in stereology and its prediction*. J Microsc 147:229–263. 10.1111/j.1365-2818.1987.tb02837.x 3430576 10.1111/j.1365-2818.1987.tb02837.x

[CR12] Hollenhorst MI, Richter K, Fronius M (2011) Ion transport by pulmonary epithelia. J Biomed Biotechnol. 10.1155/2011/174306 22131798 10.1155/2011/174306 PMC3205707

[CR13] Hsia CCW, Hyde DM, Ochs M, Weibel ER (2010) An Official Research Policy Statement of the American Thoracic Society/European Respiratory Society: Standards for Quantitative Assessment of Lung Structure. Am J Respir Crit Care Med 181:394–418. 10.1164/rccm.200809-1522ST 20130146 10.1164/rccm.200809-1522ST PMC5455840

[CR14] Knudsen L, Brandenberger C, Ochs M (2021) Stereology as the 3D tool to quantitate lung architecture. Histochem Cell Biol 155:163–181. 10.1007/s00418-020-01927-0 33051774 10.1007/s00418-020-01927-0 PMC7910236

[CR15] Lee P, Chandel NS, Simon MC (2020) Cellular adaptation to hypoxia through hypoxia inducible factors and beyond. Nat Rev Mol Cell Biol 21:268–283. 10.1038/s41580-020-0227-y 32144406 10.1038/s41580-020-0227-y PMC7222024

[CR16] Massaro GD, Gail DB, Massaro D (1975) Lung oxygen consumption and mitochondria of alveolar epithelial and endothelial cells. J Appl Physiol 38:588–592. 10.1152/jappl.1975.38.4.588 1141087 10.1152/jappl.1975.38.4.588

[CR17] Mercurio AR, Rhodin JAG (1984) An electron microscopic study on the type I pneumocyte in the cat: postnatal morphogenesis. J Morphol 182:169–178. 10.1002/jmor.1051820205 6512860 10.1002/jmor.1051820205

[CR18] Mühlfeld C, Mayhew TM, Gehr P, Rothen-Rutishauser B (2007) A novel quantitative method for analyzing the distributions of nanoparticles between different tissue and intracellular compartments. J Aerosol Med Depos Clear Eff Lung 20:395–407. 10.1089/jam.2007.0624 10.1089/jam.2007.0624 18158712

[CR19] Ochs M, Mühlfeld C (2013) Quantitative microscopy of the lung: a problem-based approach. Part 1: basic principles of lung stereology. Am J Physiol Cell Mol Physiol 305:L15–L22. 10.1152/ajplung.00429.2012 10.1152/ajplung.00429.2012 23624789

[CR20] Ochs M, Nyengaard JR, Jung A et al (2004) The number of alveoli in the human lung. Am J Respir Crit Care Med 169:120–124. 10.1164/rccm.200308-1107OC 14512270 10.1164/rccm.200308-1107OC

[CR21] Ochs M, Knudsen L, Hegermann J et al (2016) Using electron microscopes to look into the lung. Histochem Cell Biol 146:695–707. 10.1007/s00418-016-1502-z 27688057 10.1007/s00418-016-1502-z

[CR22] Schindelin J, Arganda-Carreras I, Frise E et al (2012) Fiji: an open-source platform for biological-image analysis. Nat Methods 9:676–682. 10.1038/nmeth.2019 22743772 10.1038/nmeth.2019 PMC3855844

[CR23] Schittny JC (2017) Development of the lung. Cell Tissue Res 367:427–444. 10.1007/s00441-016-2545-0 28144783 10.1007/s00441-016-2545-0 PMC5320013

[CR24] Schmid L, Hyde DM, Schittny JC (2023) Microvascular maturation of the septal capillary layers takes place in parallel to alveolarization in human lungs. Am J Physiol—Lung Cell Mol Physiol 325:L537–L541. 10.1152/ajplung.00425.2022 37605833 10.1152/ajplung.00425.2022 PMC11068427

[CR25] Schneider JP, Wrede C, Hegermann J, et al (2018a) The human alveolar epithelial type I cell reconstructed in 3D - more than a simple squamous cell. In: 7th DZL Annual Meeting. Gießen, p 298

[CR26] Schneider JP, Wrede C, Hegermann J, et al (2018b) Human alveolar epithelial type I cells reconstructed in 3D—more than simple squamous cells. In: 113th Annual Meeting of the Anatomische Gesellschaft. Rostock, p P32

[CR27] Schneider JP, Wrede C, Hegermann J et al (2019) On the topological complexity of human alveolar epithelial type 1 cells. Am J Respir Crit Care Med 199:1153–1156. 10.1164/rccm.201810-1866LE 30758981 10.1164/rccm.201810-1866LE

[CR28] Schneider JP, Wrede C, Mühlfeld C (2020) The three-dimensional ultrastructure of the human alveolar epithelium revealed by focused ion beam electron microscopy. Int J Mol Sci 21:1089. 10.3390/ijms21031089 32041332 10.3390/ijms21031089 PMC7038159

[CR29] Schneider JP, Hegermann J, Wrede C (2021) Volume electron microscopy: analyzing the lung. Histochem Cell Biol 155:241–260. 10.1007/s00418-020-01916-3 32944795 10.1007/s00418-020-01916-3 PMC7910248

[CR30] Shirasawa M, Fujiwara N, Hirabayashi S et al (2004) Receptor for advanced glycation end-products is a marker of type I lung alveolar cells. Genes Cells 9:165–174. 10.1111/j.1356-9597.2004.00712.x 15009093 10.1111/j.1356-9597.2004.00712.x

[CR31] Sterio DC (1984) The unbiased estimation of number and sizes of arbitrary particles using the disector. J Microsc 134:127–136. 10.1111/j.1365-2818.1984.tb02501.x 6737468 10.1111/j.1365-2818.1984.tb02501.x

[CR32] Tschanz SA, Burri PH, Weibel ER (2011) A simple tool for stereological assessment of digital images: the STEPanizer. J Microsc 243:47–59. 10.1111/j.1365-2818.2010.03481.x 21375529 10.1111/j.1365-2818.2010.03481.x

[CR33] van Steensel B, van Binnendijk EP, Hornsby CD et al (1996) Partial colocalization of glucocorticoid and mineralocorticoid receptors in discrete compartments in nuclei of rat hippocampus neurons. J Cell Sci 109:787–792. 10.1242/jcs.109.4.787 8718670 10.1242/jcs.109.4.787

[CR34] Vidić B, Burri PH (1983) Morphometric analysis of the remodeling of the rat pulmonary epithelium during early postnatal development. Anat Rec 207:317–324. 10.1002/ar.1092070210 6650864 10.1002/ar.1092070210

[CR35] Weibel ER (1971) The mystery of “non-nucleated plates” in the alveolar epithelium of the lung explained. Acta Anat (Basel) 78:425–443. 10.1159/000143605 4995841 10.1159/000143605

[CR36] Weibel ER (1973) Morphological basis of alveolar-capillary gas exchange. Physiol Rev 53:419–495. 10.1152/physrev.1973.53.2.419 4581654 10.1152/physrev.1973.53.2.419

[CR37] Weibel ER (2015) On the tricks alveolar epithelial cells play to make a good lung. Am J Respir Crit Care Med 191:504–513. 10.1164/rccm.201409-1663OE 25723823 10.1164/rccm.201409-1663OE

[CR38] Weibel ER (2017) Lung morphometry: the link between structure and function. Cell Tissue Res 367:413–426. 10.1007/s00441-016-2541-4 27981379 10.1007/s00441-016-2541-4

[CR39] Weibel ER, Gil J (1968) Electron microscopic demonstration of an extracellular duplex lining layer of alveoli. Respir Physiol 4:42–57. 10.1016/0034-5687(68)90006-6 4867416 10.1016/0034-5687(68)90006-6

[CR40] Zeltner TB, Burri PH (1987) The postnatal development and growth of the human lung. II Morphology Respir Physiol 67:269–282. 10.1016/0034-5687(87)90058-2 3575906 10.1016/0034-5687(87)90058-2

[CR41] Zeltner TB, Caduff JH, Gehr P et al (1987) The postnatal development and growth of the human lung. I Morphometry Respir Physiol 67:247–267. 10.1016/0034-5687(87)90057-0 3575905 10.1016/0034-5687(87)90057-0

